# Cancer du sein et grossesse: à propos de 15 cas colligés au centre de maternité de Monastir, Tunisie

**DOI:** 10.11604/pamj.2021.38.180.23108

**Published:** 2021-02-17

**Authors:** Ahmed Hajji, Houda El Mhabrech, Amira Daldoul, Nada Toumia, Awatef Hajjaji, Manel Njima, Fethi Jebali, Raja Faleh

**Affiliations:** 1 Service de Gynécologie Obstétrique, Centre de Maternité et de Néonatologie de Monastir, Centre Hospitalier Universitaire Fattouma Bourguiba Monastir, Université de Monastir, Monastir, Tunisie,; 2 Service d´Imagerie Médicale, Hôpital Hadj Ali Soua, Ksar Hellal, Université de Monastir, Monastir, Tunisie,; 3 Service de Carcinologie, Centre Hospitalier Universitaire Fattouma Bourguiba Monastir, Université de Monastir, Monastir, Tunisie,; 4 Service d´Anatomopathologie, Centre Hospitalier Universitaire Fattouma Bourguiba Monastir, Université de Monastir, Monastir, Tunisie,; 5 Service d´Anesthésie-réanimation B, Centre Hospitalier Universitaire Fattouma Bourguiba Monastir, Université de Monastir, Monastir, Tunisie

**Keywords:** Cancer du sein, grossesse, imagerie, chirurgie, chimiothérapie, radiothérapie, Breast cancer, pregnancy, imaging, surgery, chemotherapy, radiotherapy

## Abstract

Le but de cette étude était de décrire les caractéristiques cliniques, radiologiques, histologiques et thérapeutiques du cancer du sein diagnostiqué au cours de la grossesse. Nous avons effectué la revue de tous les cancers du sein diagnostiqués au cours de la grossesse dans le centre Maternité et de Néonatologie de Monastir -Tunisie, sur une période allant de 2004 à 2019. Nous avons ainsi colligé 15 cas. L'âge moyen des patientes était de 34 ans. La plupart des cancers du sein associés à la grossesse (CSAG) était diagnostiquée en post-partum. Le carcinome canalaire infiltrant était le type histologique majeur (93% de cas), un cas rare de carcinome sécrétoire a été observé. Le stade clinique était dominé par les formes T2 et T4. Les récepteurs hormonaux étaient négatifs dans 40% des cas, le récepteur HER2 était positif dans 26,6% des cas. Le traitement incluait la chirurgie, radiothérapie, chimiothérapie et palliative. La moyenne de survie est de 32,2 mois. Le cancer du sein associé à la grossesse est une entité rare. Son pronostic est globalement mauvais en raison de l'âge jeune de survenue et d´un diagnostic souvent tardif. La décision thérapeutique constitue une contrainte difficile, par nécessité, multidisciplinaire à laquelle participera la patiente. La thérapie ciblée reste le grand espoir des nouvelles thérapies.

## Abstract (en)

The purpose of this study was to describe the clinical, radiological, histological and therapeutic features of breast cancer diagnosed during pregnancy. We performed a review of all medical records of patients with breast cancer diagnosed during pregnancy in the Maternity and Neonatal Center, Monastir-Tunisia, over the period 2004-2019. We collected data on 15 cases. The average age of patients was 34 years; most pregnancy-associated breast cancers (PABCs) had been diagnosed during the postpartum period. Invasive ductal carcinoma was the major histological type (93% of cases), a rare case of secretory breast carcinoma had been observed. The main clinical stages were T2 and T4 breast cancer. Hormone receptor (HR)-negative breast cancers had been reported in 40% of cases, HER2-positive breast cancers in 26.6% of cases. Treatment included surgery, radiotherapy, chemotherapy and palliative chemotherapy. The median overall survival was 32.2 months. Pregnancy-associated breast cancer is a rare entity. Patients’ prognosis is generally poor due to the young age at onset and a usually delayed diagnosis. Patients should participate in therapeutic decision making, which is difficult and multidisciplinary. Targeted therapy is the great hope for new therapies.

## Introduction

Le cancer du sein constitue un problème majeur de santé publique, c´est le cancer féminin le plus fréquent en Tunisie et dans le monde. L´association d´un cancer du sein à une grossesse est un évènement rare, sa fréquence est de 1/3000 à 1/10000 grossesses [1, 2]. Elle est définie par la survenue d´un cancer du sein pendant la grossesse ou durant l´année suivant l´accouchement. Le cancer du sein associé à la grossesse (CSAG) est souvent décrit comme un cancer plus agressif parce qu´il est détecté plus tardivement qu´en dehors de grossesse [3, 4]. La survenue concomitante de ces deux entités cliniques pose différents problèmes d´ordre diagnostique, thérapeutique et pronostique. L´objectif de notre travail est de décrire les particularités cliniques et radiologiques ainsi que les modalités de prise en charge d'un cancer du sein au cours de la grossesse.

## Méthodes

Il s´agit d´une étude rétrospective mono centrique colligeant 15 patientes chez qui un cancer du sein était diagnostiqué au cours de la grossesse ou une année en post partum sur une période de 16 ans (2004-2019) et prises en charge au centre de Maternité et Néonatologie de Monastir-Tunisie. Pour tous ces cas, le diagnostic était suspecté à l´examen clinique ou l´imagerie et confirmé par l´examen anatomopathologique après microbiopsie écho-guidée ou exérèse chirurgicale. Toutes nos patientes étaient traitées et/ou opérées au service de Gynécologie-Obstétrique et de Carcinologie du centre.

## Résultats

**Figure 1:**
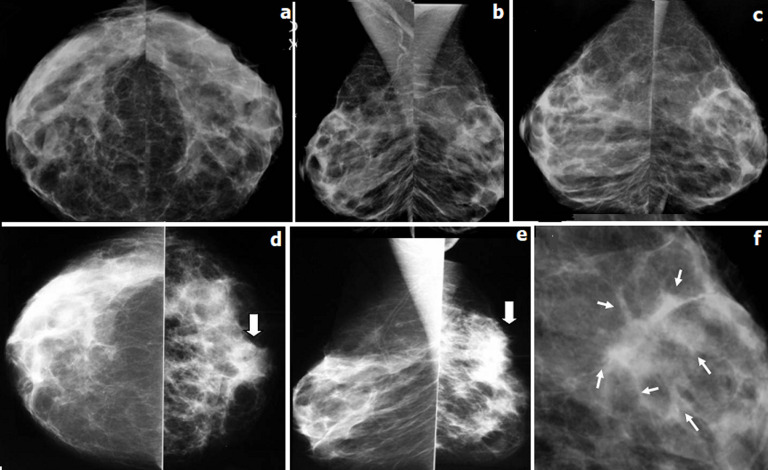
patiente âgée de 38 ans, G4P2A2, sans antécédents personnels ou familiaux de pathologies mammaires; mammographie bilatérale en incidence de face (a), oblique externe (b) et profil (c) était réalisée en avril 2013, interprétée comme normale et étant classée ACR 1; mammographie bilatérale en incidence de face (d), oblique externe (e) réalisée un an après, à 6 SA, (f): zoom sur le QSEG de la mammographie d´avril 2013: (d, e): asymétrie de taille des seins avec un sein gauche de petite taille siège à l´union des quarants supérieurs d´une masse à contours estompés; (f): la relecture de la mammographie d´avril 2013: distorsion architecturale d´aspect stellaire émettant des spicules courts et épais (flèches)

**Figure 2:**
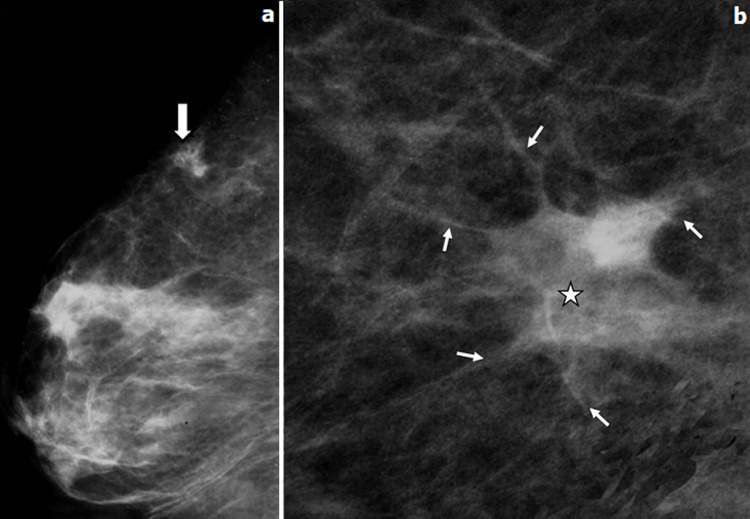
patiente de 37 ans, G4 P3 A0, l´histoire de la maladie remonte à juin 2011 où la patiente a découvert un nodule de sein droit douloureux à la palpation alors qu´elle était enceinte sur un terme de 16 SA; dans ses antécédents, sa tante maternelle est décédée d´un cancer du sein; mammographie unilatérale droite en incidence oblique externe (a), un zoom sur le QSED (b): masse stellaire (flèche épaisse) qui envoie des spicules long (flèches), ayant un centre dense (étoile)

**Figure 3:**
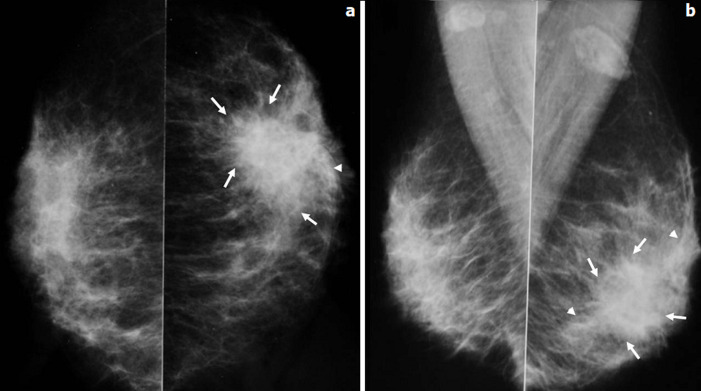
patiente âgée de 40 ans, G9 P6 A3, elle consultait en avril 2012 pour nodule du sein gauche avec mastodynie sur un terme de 8 SA; mammographie bilatérale en incidence de face (a) et oblique externe (b) masse (flèches) de 3cm environ, à contours estompés émettant des prolongements en queue de comète (tête de flèche)

**Figure 4:**
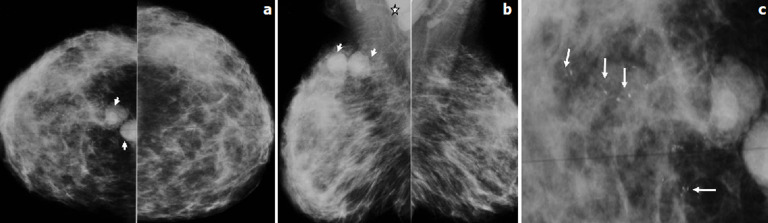
patiente âgée de 37 ans, G6 P4 A1, qui consultait en juillet 2012 pour nodule du sein droit associé à une mastodynie; le nodule était découvert alors que la patiente était enceinte à un terme de 10 SA; mammographie bilatérale en coupe axiale (a), oblique externe (b) et un zoom sur les quadrants supérieurs droits (c): deux masses arrondies contiguës (tête de flèche), à contours nets et microlobulés s´accompagnant de microcalcifications polymorphes ayant une distribution galactophorique (flèches) et un épaississement des tissus sous cutanés en rapport avec une mastite inflammatoire; ADP axillaires droite (étoile)

**Figure 5:**
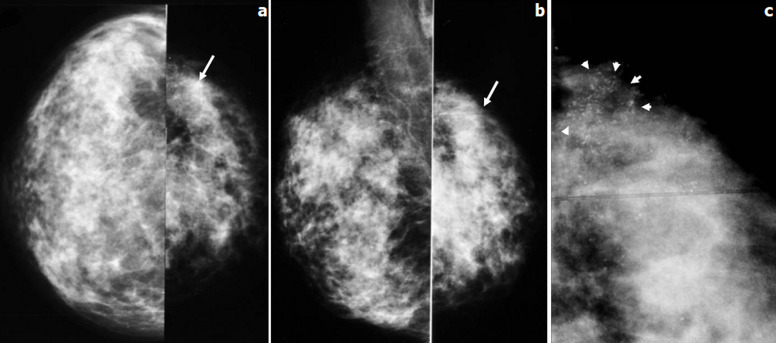
patiente âgée de 34 ans, G2P2, ayant découvert une masse du sein gauche sur un terme de 34 SA; l´examen des seins objectivait une masse mammaire gauche associée à des signes inflammatoires locaux ne permettant pas d´apprécier le volume tumoral et une adénopathie axillaire homolatérale; mammographie bilatérale en incidence de face (a) et d´oblique externe (b) et un zoom sur le QSEG (c): asymétrie de volume des seins avec un petit sein gauche; foyer de microcalcifications poudreuses et polymorphes occupant le QSEG sans masse ni distorsion architecturale visible

**Figure 6:**
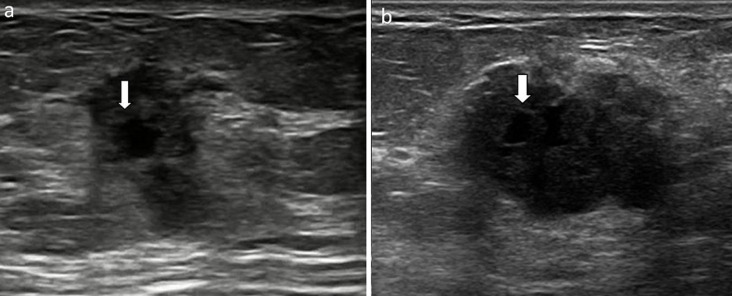
échographie mammaire réalisée chez une patiente âgée de 38 ans à 6 SA (a), et chez une patiente âgée de 28 ans, à 7 SA; (b): masses hypoéchogènes siège de zone liquidienne centrale (flèche épaisse)

Nous avons colligé 15 cas de cancer du sein associé à la grossesse parmi 364 cas de cancer du sein diagnostiqué pendant la même période; ce qui représente 5,86%. L'âge moyen des patientes au moment du diagnostic était de 34 ans. Le diagnostic était fait au cours de la grossesse dans 6 cas (40% des cas) et en post-partum dans 09 cas (60% des cas). Pour les cancers découverts au cours de la grossesse, le terme moyen au moment du diagnostic était de 22 SA. La palpation d'un nodule mammaire constitue la circonstance de découverte prédominante (60%) avec un délai moyen de consultation de 2 mois. La tumeur était bilatérale dans 2 cas. Au moment du diagnostic, la taille tumorale moyenne était de 38mm, des signes inflammatoires locaux étaient présents dans 5 cas et des adénopathies axillaires suspectes dans 2 cas. La mammographie avait montré des images suspectes en faveur de la malignité dans 73% des cas: masses à contours stellaires (5 cas) (Figure 1, Figure 2), estompés (Figure 3) et micro lobulés (Figure 4) (3 cas) associées à des microcalcifications dans 4 cas (Figure 5). Une distorsion architecturale était observée chez 6 patientes et une asymétrie focale dans 3 cas. Des signes inflammatoires locaux étaient notés dans 5 cas. La mammographie n´avait pas montré d´anomalies dans 2 cas. Dans 86 % des cas, l'échographie avait détecté un aspect en faveur de malignité: images hypo échogènes (15 cas), hétérogènes avec présence de zone liquidienne intra-lésionnelle (3 cas) (Figure 6). Les contours étaient spiculés (5 cas), micro lobulés (5 cas), polylobés (3 cas). Une atténuation postérieure des échos (10 cas), un grand axe vertical (5 cas). D´autres signes échographiques plus inhabituels étaient également notés: un renforcement postérieur des échos (2 cas) et un grand axe horizontal (10 cas) et des contours réguliers (2 cas). Au terme de ce bilan radiologique, les lésions étaient classées ACR 5 dans 9 cas, ACR 4 dans 4 cas. Deux patientes seulement étaient classées ACR 2 (1 cas) et ACR 3 (1 cas).

Une microbiopsie écho-guidée était pratiquée chez 80% de nos patientes. Le carcinome infiltrant de type non spécifique était retrouvé chez 94 % des patientes (14 cas) avec un seul cas de carcinome sécrétoire. Il s´agissait d´un grade SBR élevé (II et III) dans 86,66% des cas, associé à un envahissement ganglionnaire dans la plupart des cas (12 cas). Les récepteurs hormonaux étaient négatifs dans 6 cas (40%), le récepteur HER2 était positif dans 26,6% des cas et 40% des patientes présentaient une tumeur de type «triple négative». Une localisation métastatique était retrouvée chez 6 patientes (40%), dont 3 étaient métastatiques d´emblée. Les métastases étaient essentiellement osseuses (5 cas). Le stade clinique était dominée par les formes T2-T4 et N0-N1. Le cancer était découvert au troisième trimestre de la grossesse dans 3 cas. Ces trois patientes ont accouché par césarienne programmée avant l´entrée en travail entre 34 et 36 semaines d´aménorrhées. Chez 2 patientes, le diagnostic était réalisé pendant le 2^e^ trimestre de la grossesse. Dans 1 cas, le diagnostic était porté lors du premier trimestre et la conduite était alors une interruption de la grossesse. La conduite à tenir avait fait l´objet d´une concertation multidisciplinaire formée par des gynécologues, des radiologues, des carcinologues et des radiothérapeutes. La tumeur était jugée opérable dans 12 cas (il s´agissait d´une chirurgie première dans 6 cas). Un traitement radical était indiqué chez 8 patientes et une tumorectomie chez 4 patientes. Dans tous les cas, un curage axillaire homolatéral était réalisé. La chimiothérapie était pratiquée chez toutes les patientes. Il s´agissait d´une chimiothérapie néoadjuvante dans 6 cas (40%), une chimiothérapie adjuvante dans 7 cas (46,66%) et une chimiothérapie palliative dans 2 cas (20%). Une radiothérapie locorégionale curative était réalisée dans 11 cas. Trois de nos patientes avaient bénéficié d´une hormonothérapie adjuvante à base de tamoxifène associée à une castration dans 2 cas. Parmi les 4 patientes qui présentaient une tumeur exprimant des récepteurs à l´Herceptine, deux patientes avaient bénéficié d'un traitement par Herceptine en adjuvant pendant une durée de 1 an. L´évolution était marquée par la rechute à distance dans 2 cas, une rechute controlatérale dans 2 cas et une récidive homolatérale sur cicatrice opératoire dans un cas. La médiane de survie était de 32,2 mois.

## Discussion

Le cancer du sein associé à la grossesse est une entité rare et de découverte souvent tardive. Elle est définie par la survenue d´un cancer du sein pendant la grossesse ou durant l´année suivant l´accouchement. Sa fréquence est de 1/3000 à 1/10000 grossesses. Elle représente 0,2% à 3,8% de l´ensemble des cancers du sein contre 5,86 % retrouvé dans notre étude [1] et 0,3‰ de l´ensemble des grossesses [5] contre 0,47‰ de notre travail. Cette proportion reste faible mais tend à accroitre du fait du recul de l'âge à la première grossesse et de l'augmentation de l´incidence globale. L´âge moyen de survenu de ce cancer varie entre 31 et 35 ans et l´âge gestationnel moyen au moment du diagnostic est de 21 SA. Le diagnostic clinique de cancer du sein est difficile lors de la grossesse ou lors de l´allaitement, secondaires aux modifications anatomiques et physiologiques du parenchyme mammaire [6, 7]. Ces derniers seront responsables d´un retard diagnostic qui peut aller jusqu´à 15 mois environ [8] contre une moyenne de 3.2 mois dans notre série. Ce retard est expliqué par le changement gravidique du sein rendant plus difficile la détection d´une masse par la patiente ou lors d´un examen clinique, d´autre part à la négligence de certains praticiens de l´examen des seins, se contentant du suivi de la grossesse, et leurs réticences non justifiées à demander des examens complémentaires de dépistage potentiellement irradiant. Les circonstances de découvertes sont diverses: la perception d´une masse mammaire, un écoulement galactophorique, des signes inflammatoires locaux ou dans le cadre d´un dépistage organisé ou non. L´examen clinique pourrait révéler une masse indolore dans 80 à 90 % des cas [9]. Cependant, 80 % des masses palpées au cours de la grossesse sont bénignes. Les formes multifocales ou bilatérales sont plus fréquentes (4,6%) que chez la femme non enceinte [10]. Deux de nos patientes avaient une localisation bilatérale et l´atteinte était multifocale dans 1 cas. Des signes d´appel faisant évoquer une évolution métastatique seront aussi recherchés en particulier. Dans notre travail 20 % des patientes étaient d´emblée métastatiques.

La mammographie peut être réalisée au cours de la grossesse moyennant une protection adéquate du ventre et du pelvis [11]. Sa sensibilité varie entre 70 et 90% avec des faux négatifs allant jusqu´à 40 % [11]. Dans notre série, le taux des faux négatifs était à 13.3%. Cet examen permet de révéler la présence de masses suspectes, de microcalcifications, une distorsion architecturale ou une asymétrie focale de densité [11, 12]. En fait, une masse était observée dans 53.3% de nos cas, des signes mammographiques indirects en faveur du diagnostic étaient souvent présents : microcalcifications (33%), distorsion architecturale (6%) ou asymétrie focale (20%). Un complément échographie doit être impératif. Il permet de distinguer une masse solide d´une masse kystique détectée ou non à la mammographie [13]. Elle permet de confirmer le caractère suspect de malignité d´une masse: échostructure hypo échogène, hétérogène, atténuation postérieur des échos, un grand axe vertical [5, 14]. Cependant, nous avons pu noter dans notre étude l´association d´autres signes inhabituels qui devraient être pris en compte chez toute femme enceinte se présentant pour une masse mammaire. Il s´agit des contours réguliers d´une masse (13.3%), un renforcement postérieur des échos (13.3%), un grand axe horizontal (66.6%) ou la présence de zones liquidiennes intra-tumorales (13.3%). L´échographie mammaire permet également de guider une microbiopsie [15], elle était pratiquée chez 80% nos patientes. La place de l´IRM durant la grossesse est discutée et n´est pas recommandée de façon systématique [1]. Cependant, elle peut être réalisée en cas de suspicion de lésions multifocales en imagerie conventionnelle, en pré-thérapeutique, avant de choisir entre une chimiothérapie néo-adjuvante ou une chirurgie, en cas d´analyse difficile des lésions ou enfin lorsqu´il s´agit d´une patiente ayant déjà eu recours à un traitement chirurgical conservateur [1, 15]. Une IRM mammaire pré-thérapeutique était réalisée dans un seul cas de notre série, elle avait confirmé le caractère bifocal des tumeurs chez cette patiente. Les mêmes types histologiques de cancer du sein qu´en dehors de la grossesse peuvent être retrouvés [16, 17]. Le carcinome infiltrant de type non spécifique est le cancer le plus fréquent (75-90%), le grade SBR est le plus souvent élevé [18], retrouvé chez 86,6% de nos patientes. Les récepteurs hormonaux sont plus fréquemment négatifs qu´en dehors de la grossesse [18, 19]. Ils étaient négatifs chez 40% de nos cas. Le HER2neu est sur exprimé dans 10-25% de tous les cancers du sein [20]. Une négativité de la sur expression de l´oncogène HER2neu était retrouvée dans 73,4% des cas de notre travail. Les cancers du sein associés à la grossesse sont des cancers généralement hautement proliférant avec une expression du Ki67 supérieure à 30% [21].

L´atteinte ganglionnaire axillaire semble plus fréquente dans les CSAG. Les chiffres rapportés dans la littérature sont variables de 47% à 89% [22]. Le protocole du traitement du cancer du sein chez la femme enceinte doit être le plus proche possible de celui proposé aux femmes non enceintes [22]. Il doit tenir compte de l´âge de la grossesse au moment du diagnostic, des préférences de la patiente et du stade de la maladie. L´interruption de grossesse ne doit plus être systématique, car elle n´améliore pas le pronostic. Elle doit être prise en fonction du désir du couple et des nécessités thérapeutiques. Le geste chirurgical dépend de la classification TNM, elle consiste soit à un traitement radical ou conservateur associé ou non à un curage ganglionnaire [23]. Une chirurgie radicale était réalisée chez 8 patientes dans notre série, et un traitement conservateur chez 4 parmi elles. La chimiothérapie ne doit pas être délaissée et doit être débutée après la fin du premier trimestre et doit être évitée à un délai de 3 semaines au moins avant l´accouchement [1, 24]. Les traitements les plus documentés sont la doxorubicine, le cyclophosphamide et le 5-fluoro-uracile [25]. Une chimiothérapie était néoadjuvante chez 6 de nos patientes, adjuvante dans 7 cas et palliative dans 2 cas. Les thérapeutiques ciblées offrent un grand espoir dans l'amélioration de la prise en charge du cancer du sein. De nombreuses nouvelles cibles sont actuellement identifiées. Dans notre série les anti-Her2, essentiellement le Trastuzumab, sont utilisés. L'hormonothérapie trouve une place large dans le traitement du CCI. Elle doit être prescrite toujours en post-partum. La radiothérapie entraine des risques pour l'enfant à naître à tous les stades de la grossesse car malgré une protection abdominale, les rayons peuvent se disperser au niveau de l'utérus. D´après les recommandations internationales, la radiothérapie externe nécessaire au traitement curatif du cancer du sein est contre-indiquée pendant la grossesse compte tenu des risques fœtaux de l´irradiation [26]. Ainsi, elle devrait être reportée après l´accouchement. Douze de nos patientes avaient bénéficié d´une radiothérapie qui était palliative dans un seul cas. Les CSAG sont considérés comme de plus mauvais pronostics que les cancers du sein non associés à la grossesse.

## Conclusion

La gravité du CSAG est liée à la fréquence des formes évoluées mettant en jeux le pronostic maternel et fœtal. Le diagnostic est parfois difficile du fait des modifications anatomiques que subit la glande mammaire au cours de la grossesse. La prise en charge implique une concertation multidisciplinaire formée par des gynécologues, des radiologues, des carcinologues et radiothérapeutes. Elle doit tenir compte de la souffrance psychologique, sociale et spirituelle de la patiente et fait appel à la qualité de la relation médecin-patiente.

### Etat des connaissances sur le sujet

- Le cancer du sein associé à la grossesse est une entité rare de mauvais pronostic du fait d´un diagnostic souvent tardif et un potentiel élevé de dissémination lymphatique;
- La prise en charge thérapeutique s´affronte à la préservation ou non de la grossesse et la contre-indication de la radiothérapie au cours de la grossesse.

### Contribution de notre étude à la connaissance

- Dans notre étude, nous avons relevé des signes radiologiques inhabituels qui devraient être pris en compte chez toute femme enceinte se présentant pour une masse mammaire. Il s´agit des contours réguliers d´une masse, un renforcement postérieur des échos, un grand axe horizontal ou la présence de zones liquidiennes intra-tumorales;
- Nous soulignons l´importance d´une interprétation prudente de l´échographie mammaire chez la femme enceinte et l´intérêt du recours à d´autres examens diagnostiques au moindre doute notamment l´IRM.
